# Cervical intervertebral disc herniation treatment via radiofrequency combined with low-dose collagenase injection into the disc interior using an anterior cervical approach

**DOI:** 10.1097/MD.0000000000003953

**Published:** 2016-06-24

**Authors:** Zhi-Jian Wang, Meng-Ye Zhu, Xiao-Jian Liu, Xue-Xue Zhang, Da-Ying Zhang, Jian-Mei Wei

**Affiliations:** Department of Pain, The First Affiliated Hospital of Nanchang University, Nanchang, China.

**Keywords:** cervical intervertebral disc herniation, collagenase, radiofrequency

## Abstract

This study aimed to determine the therapeutic effect of radiofrequency combined with low-dose collagenase injected into the disc interior via an anterior cervical approach for cervical intervertebral disc herniation.

Forty-three patients (26–62-year old; male/female ratio: 31/12) with cervical intervertebral disc herniation received radiofrequency combined with 60 to 100 U of collagenase, injected via an anterior cervical approach. The degree of nerve function was assessed using the current Japanese Orthopaedic Association (JOA) scoring system at 3 and 12 months postoperation. A visual analogue scale (VAS) was used to evaluate the degree of pain preoperation and 7 days postoperation. The preoperative and 3 month postoperative protrusion areas were measured and compared via magnetic resonance imaging (MRI) and picture archiving and communication systems (PACS).

Compared with the preoperative pain scores, the 7-day postoperative pain was significantly reduced (*P* <0.01). The excellent and good rates of nerve function amelioration were 93.0% and 90.7% at 3 and 12 months postoperation, respectively, which was not significantly different. Twenty-seven cases exhibited a significantly reduced protrusion area (*P* <0.01) at 3 months postoperation. No serious side effects were noted.

To our knowledge, this is the first study to demonstrate that the use of radiofrequency combined with low-dose collagenase injection into the disc interior via an anterior cervical approach is effective and safe for the treatment of cervical intervertebral disc herniation.

## 1 Introduction

Intradiscal collagenase chemonucleolysis is an effective treatment for cervical intervertebral disc herniation.^[1–3]^ Collagenase specifically lyses collagen, which leads to the diminution or disappearance of herniation and subsequently releases the mechanical pressure of disc protrusion to the nerve.^[4]^ However, during the hydrolysis of the protrusion following chemonucleolysis, the pressure in the intervertebral disc and the spinal canal may increase, which may exacerbate the compression to the nerve root, aggravate radicular pain, or lead to other severe complications, such as compressive spinal cord injury, which have been correlated with the dose of collagenase.^[5,6]^

Radiofrequency treatment may cause focal nucleus pulposus pyknosis and decrease the intradiscal pressure. Radiofrequency treatment may also be used to repair the annulus or destroy hyperplastic migration of nerve endings within the annulus and inhibit the release of inflammatory mediators. Radiofrequency thermocoagulation has been clinically used as an approach to treat discogenic pain.^[7]^ The efficacy of clinical applications in protruded and prolapsed intervertebral disc herniation has not been extensively studied. Furthermore, there are risks of nerve root damage in protrusion targeting treatments.^[8]^

The avoidance of nerve compression aggravation caused by collagenase injection into the intervertebral disc is of substantial clinical interest. Radiofrequency may decrease the pressure caused by the dissolution of nucleus pulposus during collagenase chemonucleolysis via a reduction in the collagen-based nucleus pulposus.^[9]^ Furthermore, radiofrequency may also decrease collagenase activity in the intervertebral space and, as a consequence, inhibit functional intervertebral disc hydrolyzation. Prolapsed nucleus pulposus from the spinal canal during radiofrequency treatment may be dissolved through collagenase chemonucleolysis. Therefore, treatment that combines targeted radiofrequency thermocoagulation with collagenase chemonucleolysis may improve the safety and efficiency of microinvasive technologies for the treatment of intervertebral disc herniation.

This study aimed to analyze the effectiveness of combining radiofrequency with low-dose collagenase injection via an anterior cervical approach to treat cervical intervertebral disc herniation. The dose of collagenase was adjusted according to the protrusion shape and size, which were assessed via contrast echoes using image guidance. Radiofrequency was subsequently applied during nucleus pulposus thermocoagulation, as well as to decrease collagenase activity in the cervical interspace. We hypothesized that the novel combination therapies would provide clinical benefits without the attenuation of enzymatic activity in the protrusion and may simultaneously reduce the risk of neurological damage, which results from high pressures within the disc. Clinical observation data obtained from March 2008 to June 2012 were analyzed and reported as follows.

## 2 Methods

### 2.1 General information

Forty-three patients (31 males and 12 females) with cervical intervertebral disc herniation who were admitted to The First Affiliated Hospital of Nanchang University from March 2008 through June 2012 were included. The inclusion criteria comprised chronic, unilateral, cervical radicular neuralgia, and evidence of discogenic disease (assessed with magnetic resonance imaging [MRI]), which indicated a single-level posterolateral herniation. The exclusion criteria comprised spinal cord degeneration, bony canal stenosis, coagulopathy, uncontrolled metabolic disease, or pregnancy.

All patients (31 males and 12 females) were divided into groups according to age, sex, decompression site, and medical history. The numbers of patients with an age less than 30-year old, 30 to 45-year old, 46 to 59-year old, and more than 60 years were 4, 8, 15, and 6, respectively. The numbers of patients with a decompression site of the C4–5 disc, C5–6 disc, and C6–7 disc were 12, 26, and 5, respectively. The numbers of patients with a medical history of less than 3 months, 3 to 12 months, and more than 12 months were 9, 23, and 11, respectively.

Prior to the study, all participants were informed about the procedure and provided written informed consent. The research was executed in accordance with the principles of the Declaration of Helsinki, and the study was approved by The First Affiliated Hospital of Nanchang University Ethics Committee.

### 2.2 Procedure

Antibiotics (cefazolin sodium, 2.0 g intravenous drip, Baiyun Mountain Pharmaceutical Co, LTD, Guangzhou, China; clindamycin hydrochloride, 1.2 g intravenous drip, Hainan Shuangcheng Pharmaceuticals Co, LTD, Haikou, China) were administered half an hour prior to the operation to prevent infection. Based on the lesion location, C-arm fluoroscopy was used to determine the puncture position.

**Figure 1:**
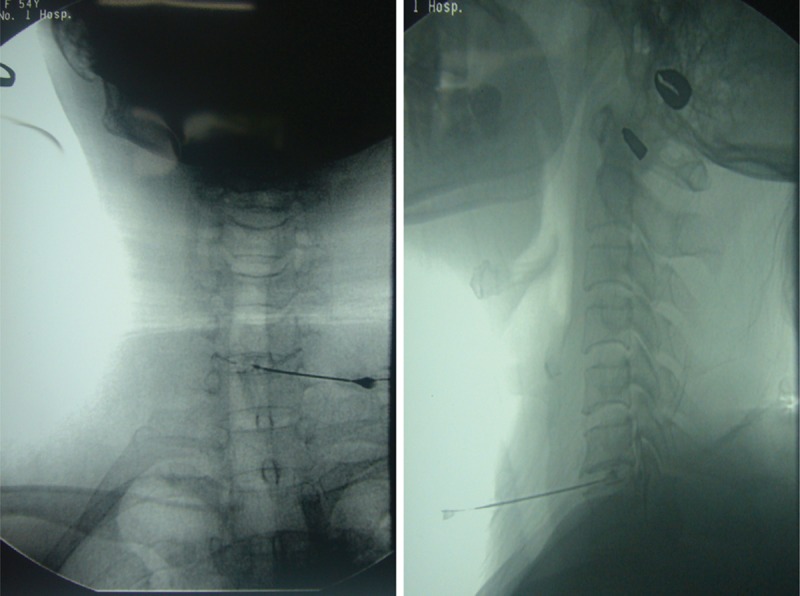
Puncture to the target site via an anterior cervical approach.

After sterilization and disinfection of the operation site, 1% lidocaine (Shanghai Zhaohui Pharmaceutical Co, LTD, Shanghai, China) was administered for local anesthesia. A radiofrequency needle (model-22G, 10-cm long, work terminal 5 mm, Elekta (Shanghai) Electa Medical Machinery Co, LTD, Stockholm, Sweden) was directed toward the basement of the protrusion using an anterior cervical approach from the contralateral side under fluoroscopic guidance (Fig. 1). When the needle was radiographically determined to be located in the basement of the protrusion, 0.2 to 0.4 mL of contrast media (iohexol, GE (Shanghai) Pharmaceutical Industry, Shanghai, China) was injected to visualize the protrusion. Then, 60 to 100 IU/0.2 to 0.4 mL of collagenase (Liaoning Weibang Biological Pharmaceutical Co, LTD, Liaoning, China) were injected. Following complete injection, electrical impedance was measured using an electrode in the cervical disc. For reference, the electrical impedance of the nucleus pulposus in the cervical intervertebral disc is 150 to 300 Ω. A sensory evoked test (100 Hz, 0.5–1.0 mA) and a motor evoked trial (2 Hz, 0.5–2.0 mA) were subsequently conducted. If these processes did not induce pain or muscle contraction, it indicated the electrode was distal to the nerve root. The initial working parameter was set to 70°C for 60 seconds to conduct single consecutive radiofrequency thermocoagulation, followed by a stepwise increase to 80°C for 30 seconds and 85°C for 60 seconds. The cervicobrachial pain and warm sensation may be duplicated, but electric shock-like numbness or pain should be avoided, which reconfirms that the electrode is localized at the target and distal to the nerve root. The parameters were subsequently set to 90°C for 90 seconds for 2 cycles. The patient was then assessed to determine neurological function and pain scores during the procedure.

The patient was returned to the ward via a flatbed after the procedure and maintained on bedrest for 7 to 10 days. Rehabilitation was provided. The patient was allowed to get up with a fixed neck brace to ensure the neck remained immobile.

### 2.3 Outcome measures

**Figure 2:**
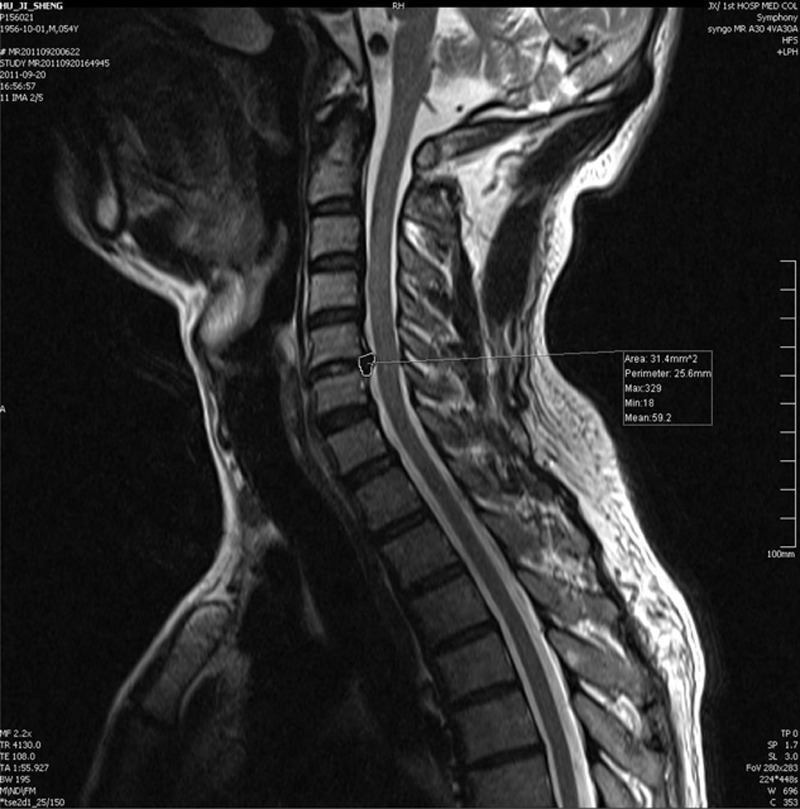
Protrusion area was measured and compared using magnetic resonance imaging and picture archiving and communication systems.

Preoperative and 7 day postoperative pain was evaluated using a visual analogue scale (VAS), in which 0 indicated no pain and 10 indicted unbearable pain. The Japanese Orthopaedic Association (JOA) Score^[10]^ was used to evaluate the recovery rate of the neurological function at 3 and 12 months after the operation. This scale consists of 6 domain scores (motor dysfunction in the upper extremities, motor dysfunction in the lower extremities, sensory function in the upper extremities, sensory function in the trunk, sensory function in the lower extremities, and bladder function), which are scored from 0 to 4, 4, 2, 2, 2, and 3, respectively. The minimum total score is 0, and the maximum total score is 17. The recovery rate was calculated using the following equation: recovery rate = [(JOA score at follow-up – preoperative JOA score)/(17 – preoperative JOA score)] × 100%. Based on the recovery rate, outcomes were classified into 4 grades: excellent (75–100%), good (50–74%), ordinary (25–49%), and poor (<25%). The preoperative and 3 month postoperative protrusion areas were measured and compared using MRI and picture archiving and communication systems (PACS; Sharp ke Asia Pacific Investment Management (Shanghai) Co, LTD, Shanghai, China). The same technical personnel performed all measurements, and the axial maximum protrusion area on the MR images was selected to calculate the area values via PACS automatic measurements (Fig. 2).

Statistical analysis was conducted using paired sample *t* tests (SPSS 11.0 software; IBM Co, Chicago, IL). A *P* value <0.05 was considered significant.

## 3 Results

The preoperative and 7 day postoperative pain scores are summarized in Tables 1–4. Compared with the preoperative scores, the pain scores were significantly decreased at 7 days postoperation in each group (*P* <0.01).

**Table 1:**
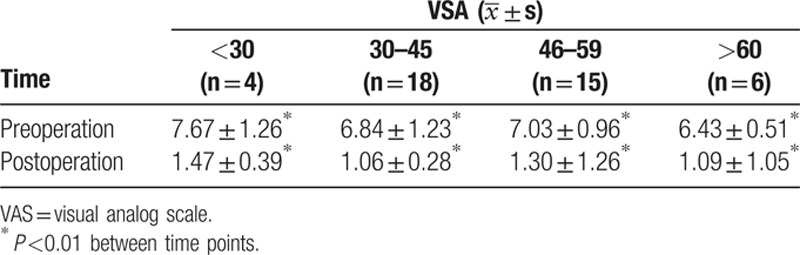
Comparison of pain degrees between preoperation and 7 days postoperation in each group according to age.

**Table 2:**
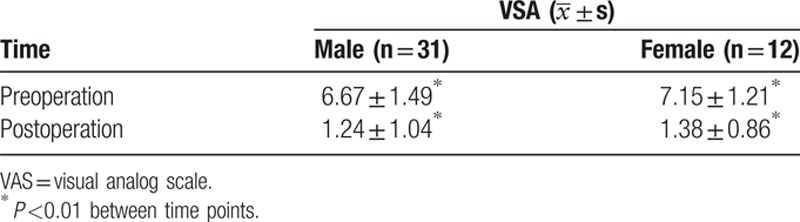
Comparison of pain degrees between preoperation and 7 days postoperation in each group according to gender.

**Table 3:**
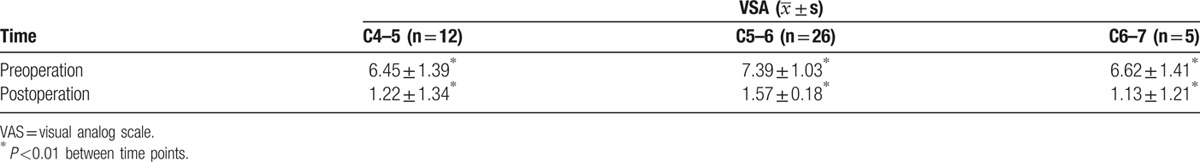
Comparison of pain degrees between preoperation and 7 days postoperation in each group according to the decompression site.

**Table 4:**
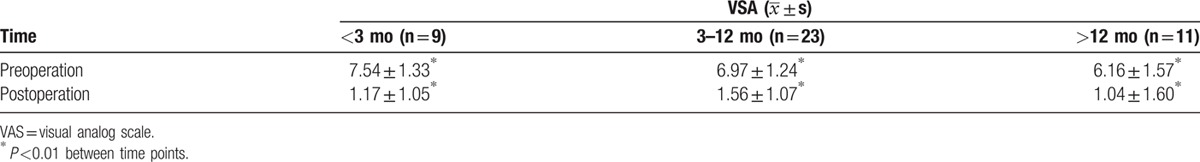
Comparison of pain degrees between preoperation and 7 days postoperation in each group according to the medical history.

**Figure 3:**
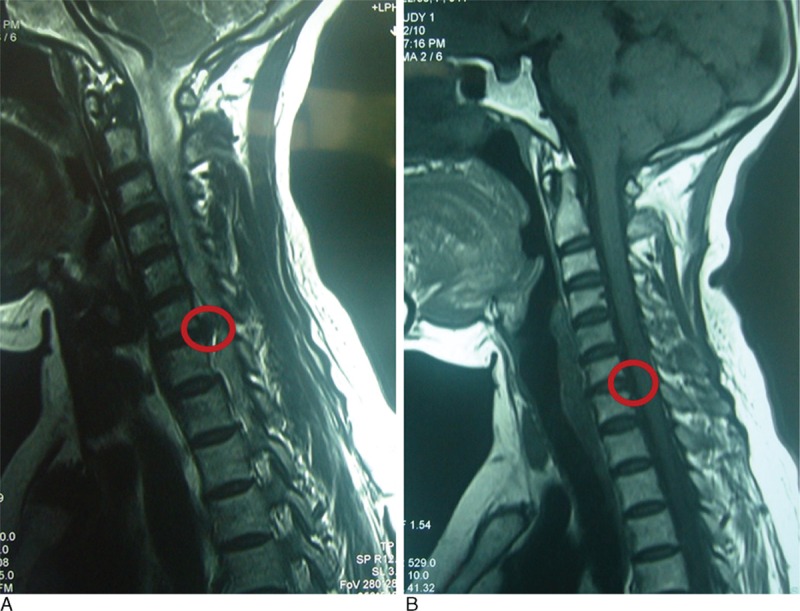
(A) Protrusion of the C6–7 disc demonstrated via a preoperative magnetic resonance imaging (MRI) scan. (B) Reduced protrusion of the C6–7 disc demonstrated via a postoperative MRI scan.

**Figure 4:**
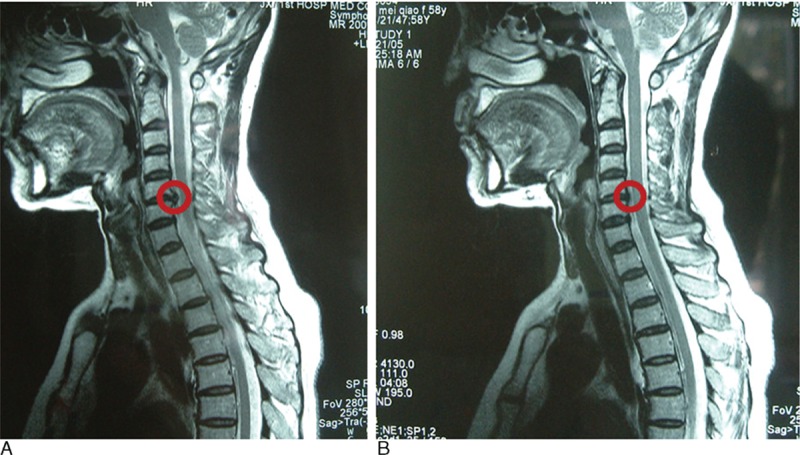
(A) Protrusion of the C5–6 disc demonstrated via a preoperative magnetic resonance imaging (MRI) scan. (B) Reduced protrusion of the C5–6 disc demonstrated via a postoperative MRI scan.

**Figure 5:**
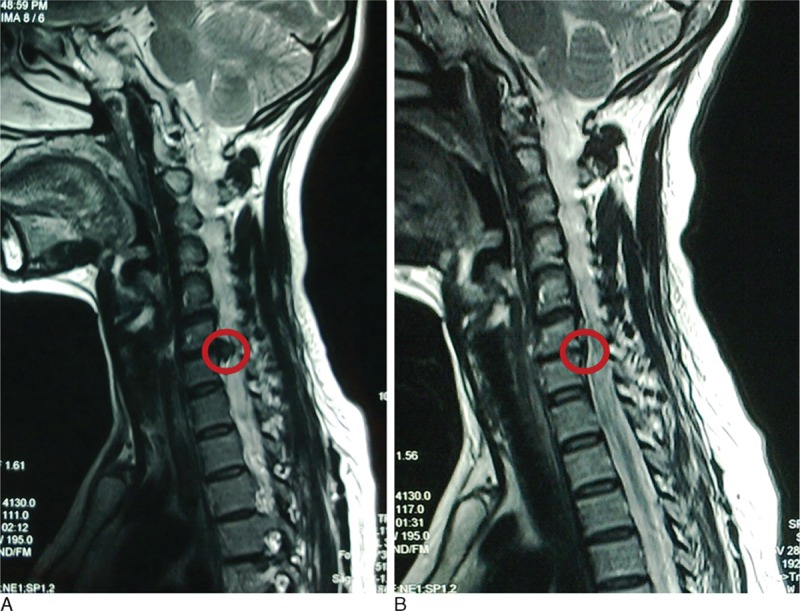
(A) Protrusion of the C6–7 disc demonstrated via a preoperative magnetic resonance imaging (MRI) scan. (B) Reduced protrusion of the C6–7 disc demonstrated via a postoperative MRI scan.

**Figure 6:**
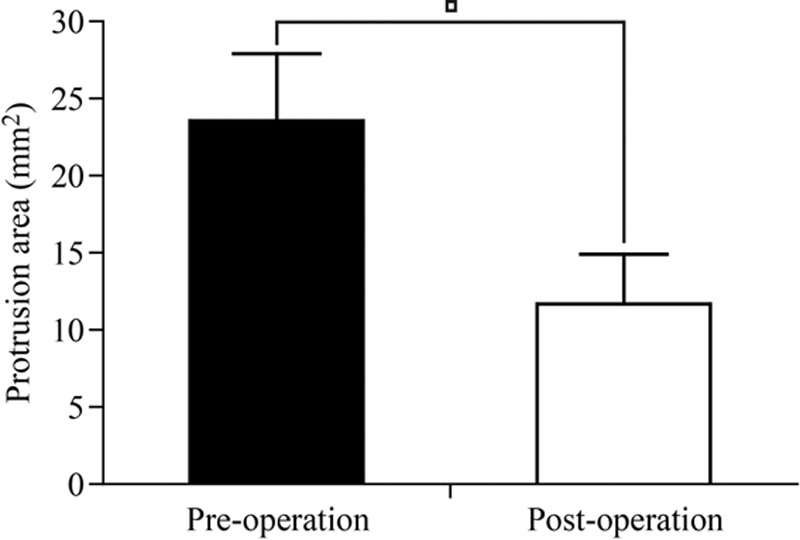
Protrusion areas at preoperation and 3 months postoperation. ^∗^*P* <0.01.

Table 5 shows the 3 and 12 month postoperative JOA scores, and the overall excellent and good recovery rates were 93.0% and 90.7%, respectively. In addition, the MRI was rechecked at 3 months postoperation in 27 patients (62.8% of all cases), and the protrusions in these cases had diminished or disappeared. Figs. 3–5 show representative MRIs before and after the procedure. The protrusion size was significantly decreased following treatment (*P* <0.01; Fig. 6). No complications or serious side effects were reported in this study.

**Table 5:**
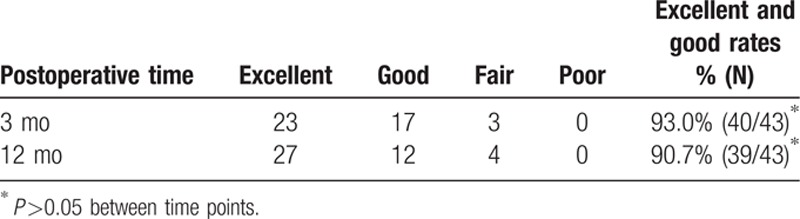
Amelioration of overall nerve function between 3 and 12 months postoperation.

## 4 Discussion

A successful treatment for intervertebral disc herniation should possess 2 essential characteristics. First, the ideal target region should comprise a protrusion herniated or prolapsed in the spinal canal, which affects the spinal nerve root. Second, the treatment protocol should minimize the impact on the structure and function of the intervertebral disc.

Previous studies have indicated that radiofrequency thermocoagulation diminished the density of the nucleus pulposus locally,^[9,11]^ and radiofrequency at the basilar region reduced the protrusion size in some patients.^[12]^ Despite the low dosage of collagenase used in this protocol, the quantity and activity of collagenase diffused into the protrusion was sufficient to act on the herniation. However, intradiscal radiofrequency would not only result in nucleus pulposus thermocoagulation and decrease the intradiscal pressure but would also decrease collagenase activity in the cervical intervertebral space, which would thus inhibit the hydrolyzation of the functional intervertebral disc. Therefore, radiofrequency thermocoagulation is beneficial to reduce the risk of neurological injury caused by the high-pressure phase following collagenase chemonucleolysis. Furthermore, it may also contribute to the protection of the intrinsic structure of the functional intervertebral disc.

Our clinical observations confirmed that radiofrequency thermocoagulation not only improved the recovery rate of patients in this study but also decreased the incidence of complications, such as nerve root injury, when small doses of collagenase (75–200 IU) were combined with target radiofrequency to treat lumbar intervertebral disc prolapse.^[13]^ The cervical intervertebral disc volume is smaller than the lumbar disc; consequently, the collagenase demand for the treatment of cervical protrusion is lower.

All patients enrolled in this study suffered a single-cervical level posterolateral herniation. After puncture via an anterior cervical approach from the contralateral side under image guidance, low-dose collagenase (60–100 IU) was intradiscally injected according to contrast echoes in the intervertebral disc. A radiofrequency electrode was subsequently inserted. Thermocoagulation was administered as soon as the electrode tip was confirmed to be located at the basilar region of the protrusion and distal to the nerve root, which was determined using sensory and motor evoked tests, as well as low-temperature radiofrequency stimulation. With respect to clinical outcomes, this therapeutic protocol reduced the protrusion size and produced short-term relief of pain symptoms. In addition, the short-mid-term neurological function recovery rate improved to a satisfactory level.

Despite these promising findings, several limitations must be considered in the interpretation of our results. For example, the long-term curative effect of this combination therapy and other potential issues, such as changes in intervertebral disc height and degeneration of adjacent intervertebral discs, were not systematically determined and require additional investigation.

To the best of our knowledge, this study provides the first evidence for the safe and effective treatment of cervical intervertebral disc herniation using radiofrequency combined with small-dose collagenase injection via an anterior cervical approach. The long-term curative effect of this combination therapy and other potential issues, such as changes in intervertebral disc height and degeneration of adjacent intervertebral discs, require additional investigation.
